# Intracranial *V. cholerae* Sialidase Protects against Excitotoxic Neurodegeneration

**DOI:** 10.1371/journal.pone.0029285

**Published:** 2011-12-15

**Authors:** Anandh Dhanushkodi, Michael P. McDonald

**Affiliations:** Departments of Neurology and Anatomy & Neurobiology, University of Tennessee Health Science Center, Memphis, Tennessee, United States of America; Weizmann Institute of Science, Israel

## Abstract

Converging evidence shows that GD3 ganglioside is a critical effector in a number of apoptotic pathways, and GM1 ganglioside has neuroprotective and noötropic properties. Targeted deletion of GD3 synthase (GD3S) eliminates GD3 and increases GM1 levels. Primary neurons from GD3S−/− mice are resistant to neurotoxicity induced by amyloid-β or hyperhomocysteinemia, and when GD3S is eliminated in the APP/PSEN1 double-transgenic model of Alzheimer's disease the plaque-associated oxidative stress and inflammatory response are absent. To date, no small-molecule inhibitor of GD3S exists. In the present study we used sialidase from *Vibrio cholerae* (VCS) to produce a brain ganglioside profile that approximates that of GD3S deletion. VCS hydrolyzes GD1a and complex b-series gangliosides to GM1, and the apoptogenic GD3 is degraded. VCS was infused by osmotic minipump into the dorsal third ventricle in mice over a 4-week period. Sensorimotor behaviors, anxiety, and cognition were unaffected in VCS-treated mice. To determine whether VCS was neuroprotective *in vivo*, we injected kainic acid on the 25th day of infusion to induce *status epilepticus*. Kainic acid induced a robust lesion of the CA3 hippocampal subfield in aCSF-treated controls. In contrast, all hippocampal regions in VCS-treated mice were largely intact. VCS did not protect against seizures. These results demonstrate that strategic degradation of complex gangliosides and GD3 can be used to achieve neuroprotection without adversely affecting behavior.

## Introduction

**Figure 1:**
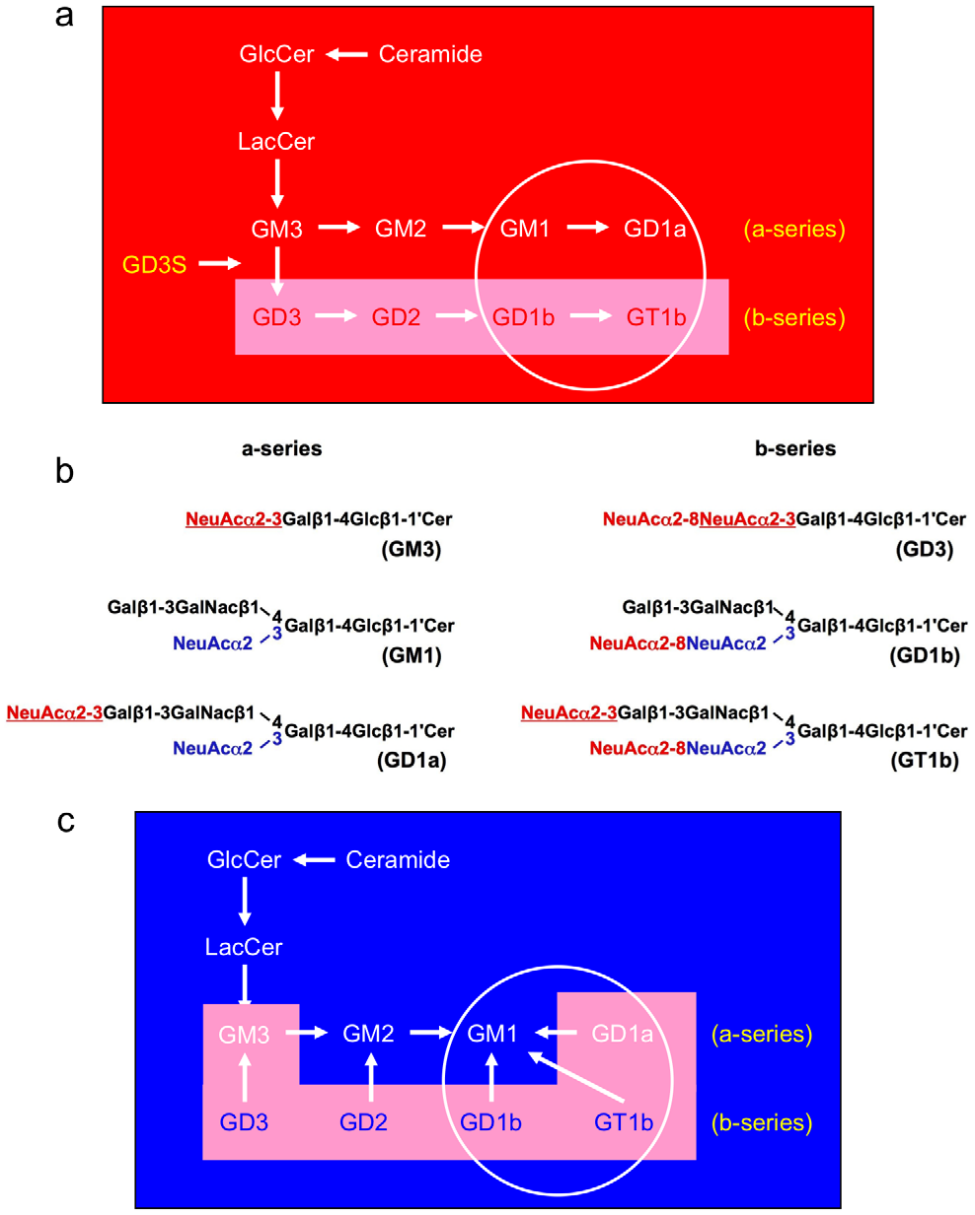
Effects of *V. cholerae* sialidase (VCS) and GD3S deletion on ganglioside biosynthesis and hydrolysis. (**a**) The ganglioside biosynthetic pathway; the four major brain gangliosides are circled. Gangliosides are synthesized by sequential addition of sialic acid residues to a sphingosine backbone. GD3 synthase (GD3S) converts GM3 to GD3, and is ultimately responsible for synthesis of all of the b-series gangliosides. GD3S−/− mice lack the b-series gangliosides including the apoptogenic GD3 and two of the four major brain gangliosides. Levels of GM1 and GD1a are elevated in GD3S null mice, as constitutively high levels of Lac-Cer are converted to a-series rather than b-series gangliosides [15]. (**b**) *Vibrio cholerae* sialidase (VCS) hydrolyzes the sialic acid α2–8 (red) linkages, and terminal α2–3 linkages (red, underlined). Internal α2–3 linkages (blue) are unaffected by VCS. Thus GD1b, GT1b, and GD1a, are converted to GM1. In addition, the apoptogenic GD3 is degraded. (**c**) Ganglioside degradative pathway; the four major brain gangliosides are circled. VCS hydrolyzes three of the four major brain gangliosides into GM1. In addition, GD3 ganglioside is degraded. The resulting brain ganglioside profile is similar to that induced by GD3S elimination except that GD1a is also hydrolyzed and levels of GM1 ganglioside are much higher [21]. Abbreviations: Gal, galactose; Glc-Cer, glucosylceramide; Lac-Cer, UDP-galactose-glucosylceramide (lactosyl ceramide); GalNac, N-acetylgalatosamine; NeuAc, N-acetylneuraminic acid (sialic acid).

The central nervous system is enriched with glycosphingolipids that bear anionic sialic acids in the outer leaflet of the plasma membranes of cells [1]. Sialic acids are a family of nine-carbon sugars that are generally found as part of glycoconjugates, mostly as terminal components like α(2–3) or α(2–6) links to hexoses or α(2–8) links to other sialic acids [2]. Important gangliosides in the central nervous system include GM1, GD1a, GD1b, GT1b, and GD3 (Fig. 1). Among these, GD3 and GM1 have received the most attention due to their involvement in cell death and neuroprotection, respectively. The role of GD3 as a critical mediator of apoptosis induced by Fas, ceramide, and amyloid-β (Aβ) is well documented [3]–[5]. In contrast, GM1 ganglioside is neuroprotective *in vitro* and in a number of lesion models [6]–[11], and has been used therapeutically to treat patients with Parkinson's disease [12]–[14].

Given the broad range of activity of both GM1 and GD3, experimental approaches that simultaneously decrease GD3 and elevate GM1 may have additive neuroprotective effects. There are several ways to achieve this *in vivo*. Targeted deletion of *St8sia1*, the gene that codes for the ganglioside biosynthetic enzyme GD3 synthase (GD3S), eliminates GD3 and elevates levels of GM1 as the constitutively normal amount of ganglioside synthesized is converted to a-series rather than b-series gangliosides (Fig. 1a). Primary neurons lacking GD3S are resistant to cell death induced by exogenous Aβ or hyperhomocysteinemia, and *in vivo* the deletion nearly eliminates Aβ and associated neuropathology and improves memory in a mouse model of Alzheimer's disease [15]. An alternate to disrupting biosynthesis is to enhance degradation. Sialidases hydrolyze sialic acid linkages on gangliosides, and can be used to degrade complex gangliosides and GD3 while increasing GM1. Although the neuroprotective effect of sialidase has not been assessed *in vivo*, Yang et al. [16] showed that chronic peripheral infusion of a sialidase from *Clostridium perfringens* enhanced spinal axon regeneration in peripheral nerve grafts after injury. This is consistent with the known effects of exogenous GM1 on nerve repair [17].

The present study was conducted to determine whether intracranial administration of sialidase would be neuroprotective against kainate-induced lesions. We used a sialidase isolated from *Vibrio cholerae* because it produces a ganglioside profile similar to that of GD3S deletion. Specifically, *V. cholerae* sialidase (VCS) cleaves the glycosidic linkages between terminal sialic acids of complex gangliotetraose gangliosides GD1a, GD1b, and GT1b, to yield increased levels of endogenous GM1 (Fig.1b; [18]–[20]). GD3 is also hydrolyzed by VCS. Thus the primary difference between VCS-treated and GD3S-null neural tissue is that GD1a is lacking in VCS-treated tissue, resulting in greater elevations of GM1 than in tissue lacking GD3S (Fig. 1c; [15], [21]).

## Methods

### Subjects

Subjects were 18 wild-type B6C3F1/J mice (Stock #100010) obtained from Jackson Laboratories (Bar Harbor, ME). Mice were housed in an AAALAC-approved vivarium with a 12-hour light/dark cycle and free access to food and water throughout the study. All procedures were approved by the Institutional Animal Care and Use Committee permit number 1697.

### *Vibrio cholerae* sialidase (VCS)

A dose of 0.5 U/ml VCS was chosen based on data from Yang et al. [16], and dissolved in artificial cerebrospinal fluid (aCSF). The composition of aCSF was 150.0 mM Na, 3.0 mM K, 1.4 mM Ca, 0.8 mM Mg, 1.0 mM P, and 155.0 mM Cl. For each mouse receiving VCS, 2.0 µl of the enzyme was diluted in 166.0 µl of aCSF. VCS and aCSF were sterilized using a 0.2-µm filter before use. The minipump delivered 6 µl of infusate per day for 28 days, at a constant rate of 0.25 µl/hour.

### Surgery

Mice were anesthetized using a cocktail of ketamine (100 mg/kg) and xylazine (10 mg/kg), and an incision made at the midline. Osmotic mini-pumps (#1004 Alzet, Inc., Cupertino, CA) were filled with VCS (n = 10) or aCSF (n = 8) and implanted in the subscapular space. Polyethylene tubing attached to the pump traversed subcutaneously to an indwelling 30-gauge cannula terminating in the dorsal third ventricle (D3V) using coordinates for from Hof et al. [22], modified for B6C3F1 mice in pilot surgeries.

### Behavior

This was the first study of intracranial administration of VCS, and thus its effects on normal behaviors were unknown. Because our investigations are focused on the translational potential of VCS, we first wanted to ensure that the novel agent did not adversely affect normal behavior. To this end we conducted a comprehensive battery of behavioral tasks. Before surgery, mice were trained to proficiency on a battery of sensorimotor tasks including rotorod, horizontal beam, inverted screen, rope climb, wire hang, and locomotor activity, as previously described [23]–[27]. Mice were matched to treatment groups based on pre-surgical locomotor activity (distance) data and assigned to VCS or aCSF groups. Starting on the third day following surgery, mice completed a comprehensive battery of behavioral tasks to measure sensorimotor function, anxiety, and spatial learning and memory. All mice performed the tasks in the same order, as listed below.

#### Sensorimotor

Mice were first placed in commercially-available activity monitors (MED-Associates, Inc., Georgia, VT) for a 60-min. session, as previously described [23]–[33]. The activity monitors measured 27×27 cm, with 16 infrared photocell beams equally spaced in the x and y axes of the horizontal plane, 1 cm from the floor of the monitor. An additional vector of 16 photobeams was situated 5 cm above the floor to track rearing.

Balance, coordination, and agility were assessed using the rotorod, horizontal beam, and rope-climb tasks. Rotorod testing was conducted using a Rotamex-5 rotorod (Columbus Instruments, Columbus, OH). After a single practice trial, mice were trained for three trials per day for 3 consecutive days to balance on a rotating rod 3 cm in diameter. The rotation speed increased from 0 to 80 RPM over a 5-min. period. If a mouse fell within 15 s it was given a second opportunity. In some cases mice would grasp the rod and rotate around with it. In this case the time at which the first rotation occurred was noted, and latency to fall or to the first rotation was the measure of interest. The horizontal beam task required the mouse to traverse a 0.64-cm wide, 80-cm long beam. Mice were motivated by a 25-watt white light bulb at the starting platform, and reinforced with entry into a dark box on the other side of the beam. Mice were placed on the 5-cm^2^ starting platform, and latency to initiate (all four paws on the beam), latency to traverse, and number of paw slips were recorded. The rope climb involved a similar avoidance of a 25-watt white light bulb and escape into a dark box. Mice were placed on the 1.5-cm-diam. rope facing down to start the trial. Latency to turn around and latency to climb the 25 cm into the dark box were recorded.

#### Anxiety

The day after sensorimotor assessment, two commonly-used anxiety tests were conducted as previously described [15], [27], [33]–[35]. Data from both tasks were collected using macros written for the public domain software NIH Image [36]–[37]. The elevated plus maze comprised four arms, 30 cm long ×6 cm wide, elevated 40 cm off the floor. The two “closed” arms had clear acrylic walls 15 cm high. The other two arms were “open” (without walls), but had 1-mm ridges along the edge to help mice hold on without falling. Mice were placed gently in the central area (8×8 cm) at the intersection of the four arms at the beginning of the 5-min. session. An image was taken every 0.5 sec., and classified as being in open or closed arms or in the central area. Dependent measures of interest were percent closed-arm entries and time on closed arms as a percentage of time on all arms, i.e., excluding time in the central area.

Approximately 2 hours following the plus-maze test, mice were placed in the periphery of a large round open field, 92 cm in diam., made of white polyethylene with 30-cm walls. An overhead camera captured images at a rate of 2 frames per sec. for the 5-min. session, during which mice were allowed to explore freely. The position of the mouse in each frame was classified as being in one of three virtual zones of approximately equal area: the center (53.2 cm diam.), periphery (8.4 cm from the wall), and an intermediate zone (the area between the periphery and center). Latency to exit the periphery, latency to enter the center, and time in each zone were variables of interest.

#### Cognition

Spatial learning and memory were assessed in a water maze, 118 cm in diam, starting the day after anxiety testing. The water ranged from 22.5–23.0°C and was made opaque using non-toxic white tempera paint. A clear acrylic platform 10 cm in diam. was submerged 0.5 cm below the surface of the water. Water maze testing was conducted in three phases: reference memory, repeated reversals, and scopolamine challenge. First, standard reference-memory training was conducted as previously described [15], [27], [32]–[34], [37]–[40]. In this task, mice were trained to find the hidden platform using the visuo-spatial extra-maze room cues, with four massed trials per day and a 20-sec. intertrial interval (ITI). The platform location did not change during the course of training, but the starting location varied from trial to trial. Mice not finding the platform within 90 sec. were placed gently on the platform for the duration of the ITI. Swim paths were recorded and converted to swim distances, escape latencies, and search error. Search error is the cumulative distance from the platform recorded each second, and is often a more sensitive indicator of water-maze learning than traditional measures of distance and latency [27], [33], [34], [41]. Swim speed and the amount of time spent in the periphery (8 cm) of the pool were recorded as controls for non-cognitive water-maze behaviors. Seven reference-memory sessions were conducted, followed by a probe trial 24 hours later in which the platform was removed and mice were allowed to swim freely for 60 sec. The amount of time spent in each quadrant as well as distance from the former platform location were recorded. The distances from the platform were used to calculate the amount of time spent over the 10-cm diam. former platform location, as well as two larger annuli of 25 and 40 cm centered on the former platform location.

Approximately 2 hours following the probe trial, the first session of repeated reversals water-maze training was conducted, following procedures described by Savonenko et al. [42]. In this version of the task, the platform location changed every day, and mice were given 10 trials per day to find the new location. All other aspects of the task were identical to those in the reference-memory version. On the fourth daily session mice were injected with saline, followed on subsequent sessions by injections of scopolamine as described below.

### Drug preparation and administration

All drugs were dissolved in physiological (0.9%) saline and injected in a volume of 10 ml/kg of body weight. Injections of scopolamine hydrobromide, scopolamine methylbromide, and saline were given subcutaneously, and kainic acid intraperitoneally. On the fourth day of repeated-reversals testing in the water maze, a saline injection was given 30 min. before the session to ensure that reaction to the injection procedures did not adversely affect performance. Starting the following day, a dose-effect curve was established with scopolamine hydrobromide at doses of 1.0 and 3.2 mg/kg body weight injected 30 min. before the water-maze session. One daily repeated-reversals session was conducted without an injection following every scopolamine dose, to ensure that performance had returned to proficient levels. To control for peripheral anti-muscarinic activity, the quaternary control methylscopolamine (scopolamine methylbromide) was administered at a dose of 3.2 mg/kg body weight 2 days following the last scopolamine injection.

### Kainic acid injections

On the 25th day of aCSF or VCS infusion, mice were injected with kainic acid,10 mg/kg of body weight. If mice did not reach *status epilepticus* within 45 min. of the injection, an additional dose was given. Once a mouse reached *status epilepticus*, no further injections were given. This escalating regimen, adapted from Hellier et al. [43], was used to minimize mortality associated with high doses of kainic acid [44]. All mice reached *status epilepticus* after 2–4 injections, with no differences between aCSF- and VCS-treated mice. Mice were observed continuously from the first injection until seizures abated. The entire experiment was video-recorded and the number and duration of seizure bouts scored offline using the modified Racine scale [45]–[46]. The motor seizures were characterized by unilateral forelimb clonus with lordotic posture (stage III), bilateral forelimb clonus and rearing (stage IV), and bilateral forelimb clonus with rearing and falling (stage V). The motor seizures subsided gradually thereafter and were not apparent 6 hours after initial *status epilepticus*. Mice were given free access to a hydrated, nutritive gel (Transgel) and subcutaneous injections of lactated Ringer's solution (5 ml/day) for 3 days after kainate injections.

### Histology and immunohistochemistry

Mice were sacrificed 3 days following the kainic acid injections. Under brief isoflurane anesthesia mice were perfused transcardially, first with saline and then with 4% paraformaldehyde for 30 min. Brains were removed and fixed overnight in the same fixative, and then 40-µm coronal sections were taken throughout the extent of the hippocampus for histological analysis. Ganglioside changes were visualized using monoclonal antibodies targeting GD3, GD1a, GD1b, GT1b, and GM1 (G2005-66, G2004-90A, G2004-90B, & G2006-90A, US Biological, Swampscott, MA; 370696-1, Northstar Bioproducts, Cambridge, MA) and visualized using a FITC-conjugated goat anti-mouse secondary antibody (F-2761, Invitrogen, Carlsbad, CA). Although cholera toxin subunit B is often used to label GM1, it is non-specific [47]. When specific antibodies are used, GM1 expression is largely, although not exclusively, restricted to white matter in adult central nervous system [48]–[49]. To observe neurodegeneration, sections were stained with cresyl violet (3095042, Sigma, St. Louis, MO) and Fluoro-Jade C (AG325, Millipore, Billerica, MA). A rabbit anti-glial fibrillary acidic protein (GFAP) polyclonal antibody (AB5804, Millipore) was used to identify reactive astrocytes.

### Statistical analyses

Most behavioral data were analyzed using one- or two-way factorial analyses of variance (ANOVA), with treatment group (aCSF or VCS) as a between-subjects factor. Time-series data were analyzed using hierarchical linear modeling, with time as an unbalanced continuous numerical repeated measure and subject as a random nominal factor nested within treatment. Quadrant analyses from the water-maze probe trial were conducted using single-sample t-tests for each treatment group and quadrant individually. Water-maze repeated-reversals data were analyzed using mixed models, with treatment (aCSF or VCS) as a between-subjects factor and drug condition (baseline, saline, methylscopolamine, scopolamine 1.0, or scopolamine 3.2) as a repeated measure. Trials was treated as an unbalanced continuous nominal repeated measure, nested within drug condition. Subject was treated as a random factor nested within treatment group. Degrees of freedom on repeated-measures analyses were corrected for variations in sphericity using Huyn-Feldt ε. To protect against spurious Type I errors, follow-up analyses were conducted only after a significant omnibus effect, except with comparisons having specific *a priori* hypotheses. All comparisons were two-tailed with α set at 0.05.

## Results

**Figure 2:**
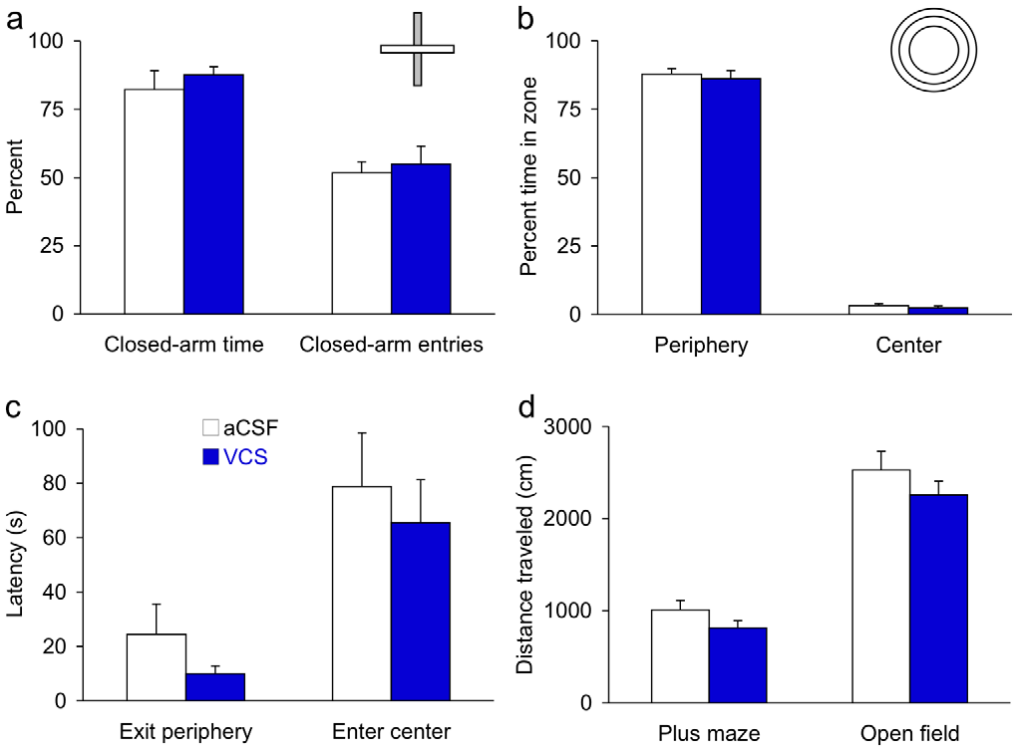
VCS does not affect locomotor activity or anxiety. Anxiety was assessed in the open-field and elevated plus maze tasks. (**a**) In the elevated plus maze, VCS- and aCSF-treated mice both spent approximately 80% of the time in the closed arms, which is normal for mice upon first exposure to the plus maze. Closed-arm entries also did not differ by treatment. (**b,c**) In the open field, VCS-and aCSF-treated mice spent the same amounts of time in the periphery, center, and intermediate zone. Their latencies to first exit the periphery and enter the central zone also did not differ. (**d**) Locomotor activity, measured by the number of cm traversed over the 5-min. session, did not differ by treatment in either the elevated plus maze or the open field.

VCS treatment did not affect exploratory locomotor activity, balance, or coordination. Distance traveled, ambulatory time, rearing, repetitive beam breaks, rope-climb latencies, and horizontal beam latencies and slips were all similar between VCS and aCSF groups [F's<1.24; p's>.298; data not shown]. VCS treatment also did not affect anxiety. Total arm entries, percent closed-arm entries, and time spent on closed arms in the plus maze were similar between groups [F's<0.64, p's>.437; Fig. 2a]. In the open field, the time spent in the peripheral and central zones, as well as the latencies to exit the periphery and enter the center, were unaffected by VCS treatment [F's<3.96, p's>.062; Fig. 2b–c]. The distances traveled in the two mazes, as well as incidence of urination and defecation, were not different between groups [F's<2.91, p's>.106; Fig. 2d].

**Figure 3:**
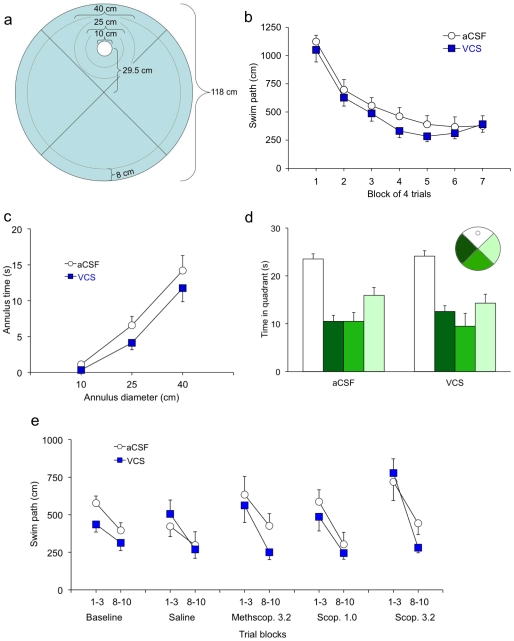
VCS does not affect spatial learning or memory. Spatial reference memory and repeated acquisition were assessed in a series of water-maze tasks. (**a**) The water maze was divided virtually into zones that allowed us to determine in which quadrant the mice swam as well as distance from the platform and time in the periphery. (**b**) Mice in both treatment groups learned to find the hidden platform proficiently. (**c, d**) Three annular zones were used to assess memory during the probe trial—10, 15, and 40 cm in diam., all of which were outside the periphery but inside the target quadrant. The 10-cm annulus represented the exact size and location of the platform during training. Chronic VCS treatment did not adversely affect spatial memory on the probe trial, either measured by the traditional quadrant divisions (**d**) or the more sensitive annular analysis (**c**). (**e**) Following the probe trial mice were re-trained to find the platform in a different location every day, in 10 trials. This repeated reversals testing did not reveal any treatment differences, either at baseline or under saline. The low dose of scopolamine did not affect learning in either group, but both groups were impaired by the 3.2 mg/kg dose. An equivalent dose of the quaternary control scopolamine methylbromide, which does not cross the blood-brain barrier, did not significantly affect performance in either group, demonstrating that the performance under scopolamine 3.2 mg/kg can be attributed to centrally-mediated cognitive impairments and not non-mnemonic performance factors.

In the water maze, both groups of mice learned to find the hidden platform within seven sessions, as indicated by decreasing swim paths over sessions [F(6,110) = 28.08, p<.0001; Fig. 3b]. There were no group differences in the facility with which spatial learning was acquired [group F(1,2) = 0.60, p = .518; Group X Session F(6,110) = 0.60, p = .726]. There were also no group differences when escape latency or search error was used to measure learning [F's<0.52, p's>.54; data not shown]. VCS treatment did not affect swim speed or peripheral swimming in the water maze [F's<1.09, p's>.376; data not shown]. On the day after the final acquisition session, mice were given a single 60-s probe trial with no platform. During this trial, treatment groups did not differ in the amount of time spent within 10, 25, or 40 cm of the center of the former platform location [group F(1,2) = 0.71, p = .490; Group X Annulus Size F(1,48) = 0.48, p = .491; Fig. 3a,c]. Quadrant analyses showed that both groups demonstrated selective search for the former platform location; mice in each group spent significantly more than chance time in the target quadrant [p's<.0001], but not in the other three quadrants [p's>.315; Fig. 3d].

Following the probe trial, mice were re-trained in the water maze but with a different platform location. For these repeated-reversals sessions, mice were given 10 60-sec trials to learn a new platform location each day. After learning this mice were challenged with the muscarinic receptor antagonist scopolamine, or appropriate controls. Five conditions were used for data analysis: baseline, saline, methylscopolamine 3.2, scopolamine 1.0, or scopolamine 3.2. The last session before the saline injection was used as the baseline. Learning was evident across all conditions, as indicated by significant improvement in performance from the first three to the last three trials [F's>5.2, p's<.03; Fig. 3e]. However, there were no significant group or Group X Trial effects [F's<3.4, p's>.088]. There were also no significant main or interaction effects across conditions, e.g., when comparing baseline vs. saline, saline vs. methylscopolamine, or methylscopolamine vs. either of the scopolamine doses [F's<1.94, p's>.170].

**Figure 4:**
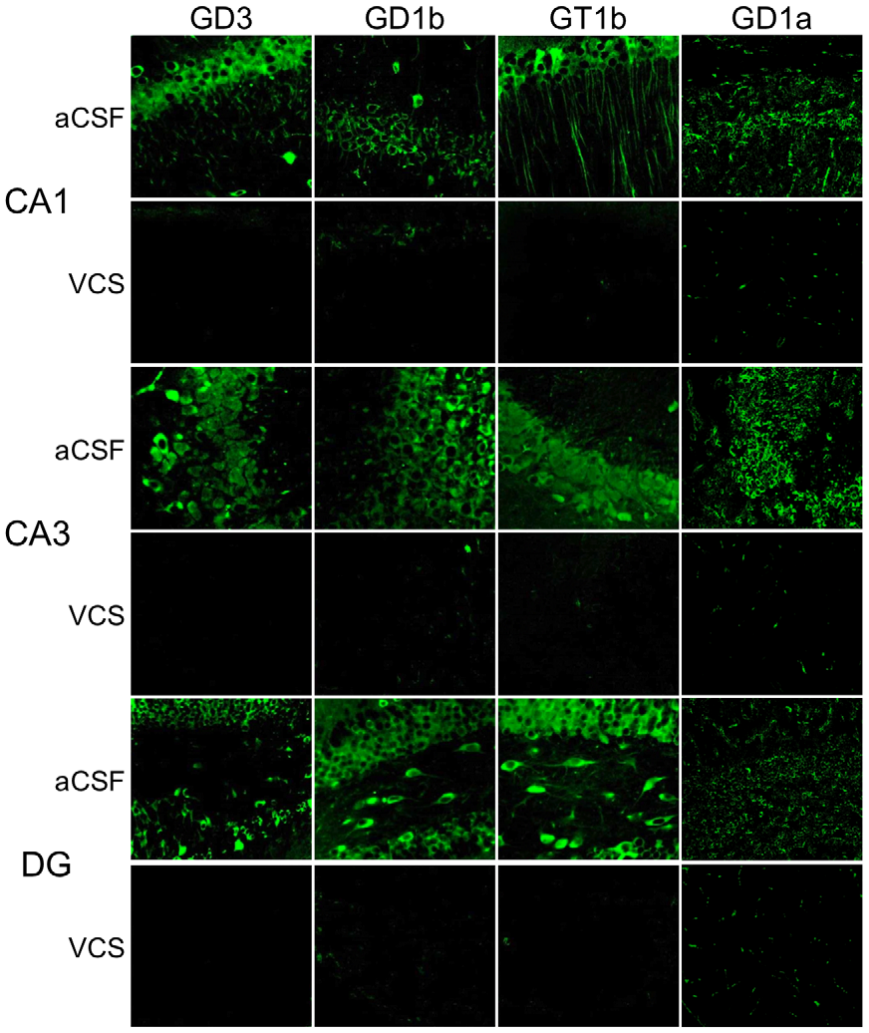
VCS infusion completely degrades GD1a and b-series gangliosides. Coronal sections were stained with antibodies to the appropriate gangliosides as described in the Methods section. VCS completely degraded three of the four major brain gangliosides (GD1a, GD1b, and GT1b) throughout the hippocampus, including the CA1 and CA3 subfields and the dentate gyrus (DG). The apoptogenic GD3 ganglioside was also hydrolyzed.

**Figure 5:**
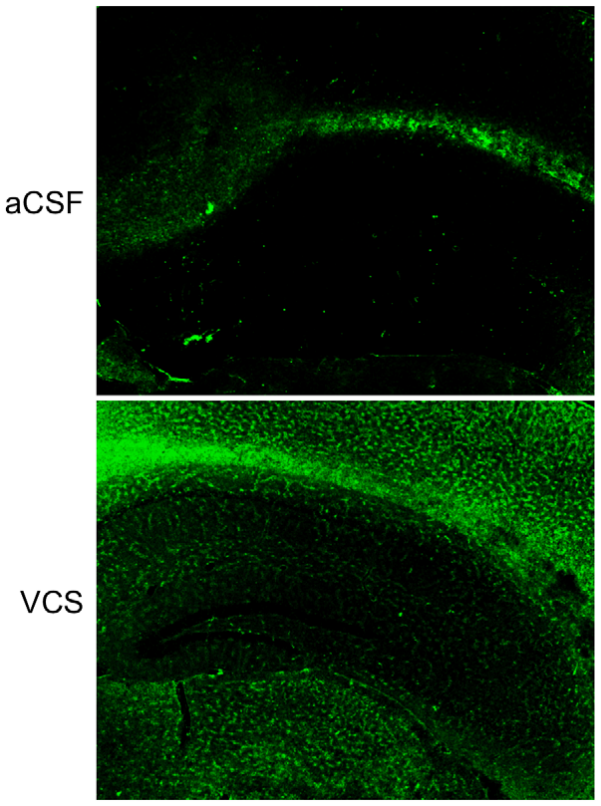
GM1 levels are significantly elevated following VCS infusion. Expression of GM1 ganglioside is largely restricted to white matter in the central nervous system, as exemplified by strong immunostaining in the corpus callosum of aCSF-treated mice. After 25 days of VCS infusion, GM1 expression was increased in white matter and the cortex, and to a lesser extent in the hippocampus.

Three days following the last water-maze session, mice were injected with kainic acid and seizure activity was observed. There was no difference between aCSF- and VCS-treated mice in the latency to reach stage III (aCSF 18.0±1.0 min.; VCS 33.0±20.1), stage IV (aCSF 70.6±15.3 min.; VCS 70.3±22.0), or stage V seizures (aCSF 87.2±12.4 min.; VCS 93.7±14.0). There was also no difference in the number of seizure bouts at stage III (aCSF 6.3±2.0; VCS 7.8±1.5), stage IV (aCSF 6.6±1.7; VCS 7.8±2.1), or stage V (aCSF 9.1±5.4; VCS 7.8±3.7). Mice were sacrificed 3 days later and brains prepared for histology and immunohistochemistry. Figure 4 shows that all hippocampal subfields in VCS-treated mice were virtually devoid of b-series gangliosides and GD1a, consistent with published reports of the *in vitro* effects of VCS on gangliosides [21]. Importantly, the apoptogenic GD3 ganglioside was also absent. In contrast, levels of GM1 ganglioside were increased after 4 weeks of VCS infusion (Fig. 5). GM1 is normally expressed al low levels in grey matter; after VCS treatment, neuronal expression of GM1 was significantly elevated, particularly in the neocortex.

**Figure 6:**
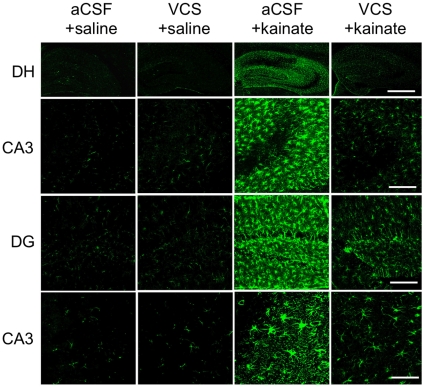
VCS prevents the neuroinflammatory response following kainic acid injection. GFAP immunofluorescence is significantly increased 3 days following kainic acid inject in aCSF-treated mice, indicating a massive inflammatory response in the hippocampus. The reactive astrogliosis was nearly absent in kainate-treated mice that had received chronic infusions of VCS.

**Figure 7:**
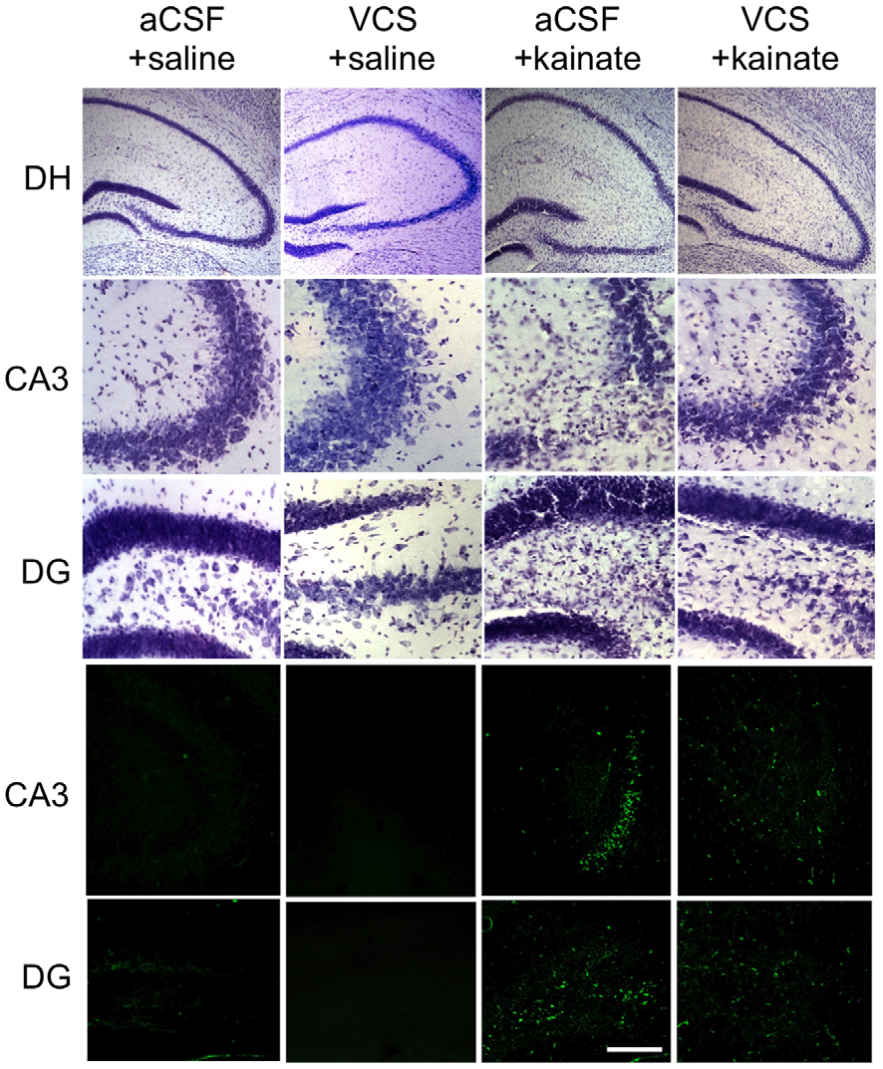
VCS is neuroprotective against kainate-induced lesions. Top panels: Cresyl-violet-stained sections of the dorsal hippocampus (DH), CA3 subfield, and dentate gyrus (DG). Robust neuronal death is largely restricted to the CA3 hippocampal subfield, and to a lesser extent in the dentate hilar region, 3 days following kainic acid injection in mice treated chronically with aCSF. In contrast, mice infused with VCS were protected from neuronal loss. Bottom panels: Fluorojade C immunofluorescence demonstrates ongoing neurodegeneration in CA3 and dentate hilar regions in aCSF-infused mice 3 days following kainic-acid injection. Kainate-injected mice treated with VCS exhibited almost no neurodegeneration.

To assess the extent of neurodegeneration and neuroinflammation 3 days following kainate injections, hippocampal tissue was stained with cresyl violet, Fluoro-Jade C, or GFAP. Figure 6 shows the dramatic neuroinflammatory response in all hippocampal subfields still evident 3 days after kainate injection in aCSF-treated mice. In contrast VCS-treated mice receiving kainic acid exhibited significantly fewer reactive astrocytes. Figure 7 shows the characteristic loss of pyramidal neurons in the CA3 region following kainate injections, in mice chronically infused with aCSF. Although kainate can induce neurodegeneration in other hippocampal regions, it is largely restricted to CA3 within 3 days of seizures. Fluorojade C staining illustrates ongoing neurodegeneration in the CA3 subfield. Some neurodegeneration is also evident in the dentate hilar region, although to a lesser extent. In contrast to aCSF-treated mice, chronic VCS infusions nearly completely prevented neuronal death in the hippocampus.

## Discussion

We have shown that 4 weeks of constant VCS infusion does not adversely affect behavior, in a comprehensive battery. All aspects of sensorimotor function, anxiety, and cognition were normal, even when challenged with scopolamine. VCS- and aCSF-treated mice also did not differ in terms of the latency to or intensity of *status epilepticus*. However, VCS-treated mice were almost completely protected against the kainate-induced neuroinflammatory response, and destruction of neurons in the CA3 and dentate hilar hippocampal subfields.

This is the first report of VCS being administered intracranially. Investigation of VCS as a potential neuroprotective agent is warranted by reports of *in vitro* neuroprotection in primary neurons lacking GD3S [15], which have a ganglioside profile similar to that of VCS (Fig. 1). After 28 days of VCS infusion, b-series gangliosides and GD1a were completely hydrolyzed in the hippocampus (Fig. 4). It is counter-intuitive to think that mice will function normally with such a substantial loss of gangliosides, including three of the four major brain gangliosides. However, we demonstrated previously that mice lacking GD3S exhibit normal cognition, anxiety, and motor function despite a complete lack of two of the four major brain gangliosides [15]. Importantly, we showed in that paper that total ganglioside and total sialic acid were unchanged in GD3S knockout mice because of increased levels of GM1 and GD1a. It is plausible that the increased a-series ganglioside compensated for some of the functions of the missing b-series gangliosides. Similarly, GM1 levels are significantly elevated in VCS-treated mice, even more so than in GD3S knockout mice due to the hydrolysis of GD1a to GM1. The excess GM1 may compensate sufficiently for GD1a, GD1b, and GT1b to ensure proficient behavioral and cognitive function. This type of functional substitution across brain gangliosides has been demonstrated *in vitro* and *in vivo* in other ganglioside knockout mice [50]–[53].

Although our data suggest that there may be significant functional compensation when brain ganglioside distribution is altered by VCS, Figures 6 and 7 demonstrate that these changes are not without effect. More than the other three major brain gangliosides, GM1 has long been considered to have broad neuroprotective properties [54]–[59]. Exogenously-administered GM1 attenuates lesions induced by, for example, ischemia, X-radiation, glutamate, 6-hydroxydopamine, and ethanol [60]–[63]. A number of neuroprotective mechanisms have been associated with GM1, including increased secretion of neurotrophic factors and activation of TrkB, and inhibition of calcium influx [64], [65]. GM1 has been shown to mimic the effects of neurotrophins, and synergize their activity, both in preventing excitotoxicity and restoring neurite outgrowth [66]–[67]. Bachis et al. [57] showed that GM1 protected primary neurons from glutamate excitotoxicity and prevented caspase-3 activation through phosphorylation of TrkB receptors. Similarly, GM1 has been shown to block the excitotoxic effect of kainate by preventing the activation and translocation of calcium-dependent protein kinase-C (PKC) and proteases [68]. Even a brief seizure bout activates more than 1,500 genes and initiates cascades that can permanently alter hippocampal circuitry [44], [69]. The initial necrotic events include increase of intracellular calcium, activation of NMDA receptors and voltage-gated calcium channels, and the release of calcium from intracellular stores following activation of metabotropic glutamate receptors [70]. Subsequently, elevated calcium levels initiate apoptotic events as evidenced by activated caspase-3, fragmented DNA, and preserved mitochondrial ultrastructural integrity and energy metabolism [71]. Thus is it likely that the persistently elevated GM1 induced by chronic VCS infusion protected against excitotoxicity by inhibiting calcium influx in kainate-injected mice.

In contrast to the putative beneficial effects of GM1 ganglioside, GD3 ganglioside is neurotoxic [3]–[5], [72]–[73]. Converging evidence implicates GD3 ganglioside as a downstream mediator of apoptosis. GD3 is synthesized *de novo* in response to Fas ligand and ceramide, and is necessary for apoptosis induced by these initiators [3], [72]–[75]. Kristal and Brown [76] reported that GD3 is both necessary and sufficient to propagate the Fas-mediated pathway, and Copani et al. [77] showed that GD3 is required for neuronal death induced by amyloid-β (Aβ). GD3 decreases the mitochondrial transmembrane potential and is upregulated in response to a number of caspases; however, its induction of apoptosis is capsase- and calcium-independent [3], [76]. Although GD3 and other b-series gangliosides were thought to be necessary for neuronal differentiation, GD3S−/− embryonic stem cells undergo normal differentiation [53]. Consistent with this, GD3S knockout mice appear normal and have a normal life span with no overt neurological or behavioral abnormalities [15]. We have shown that primary neurons from GD3S−/− pups are resistant to cell death induced by 10 µM Aβ or hyperhomocysteinemia induced by folate deficiency [15]. Like GD3S-null mice, VCS-treated mice in the present study have a complete lack of GD3 ganglioside in the neuronal layers of the hippocampal subfields, including CA3 (Fig. 4). This suggests that GD3 may be necessary for kainate-induced cell death *in vivo*. We do not know whether the lack of GD3 or calcium inhibition afforded by elevated GM1 was responsible for the neuroprotective properties of VCS, or if they both played a role. Both changes occur simultaneously and immediately upon exposure to VCS. Teasing out the relative contribution of these two putatively neuroprotective events may be difficult, because reagents do not exist that will specifically affect one or the other without simultaneously affecting other gangliosides, and exogenous gangliosides do not necessarily behave like endogenous gangliosides. Taken together, these results demonstrate that VCS induces a neuroprotective ganglioside profile without altering normal behaviors.

The effects of kainate in the present study and in Wu et al. [6] suggest that GM1 can take over many functions of the other gangliosides when they are missing. This confirms and extends our previous reports showing neuroprotection in primary neurons lacking GD3S, and normal behavior in GD3S knockout mice [15]. The robust neuroprotection in the present study demonstrates in principle that degradation of brain gangliosides to increase GM1 and eliminate GD3 has potential therapeutic benefit without significant adverse behavioral effects. Given the importance of GD3 ganglioside at the convergence of multiple cell-death pathways, this approach may have broad applicability, and further exploration VCS, and more efficient delivery systems, is warranted.
